# Supplementary material for: Data science approaches provide a roadmap to understanding the role of abscisic acid in defence
Source: Quant Plant Biol. 2023 Feb 8;4:e2. doi: 10.1017/qpb.2023.1 (PMC10095806; doi:10.1017/qpb.2023.1)
Supplement: Supplementary file 1 [file S2632882823000012sup001.zip › S2632882823000012sup001.pdf]

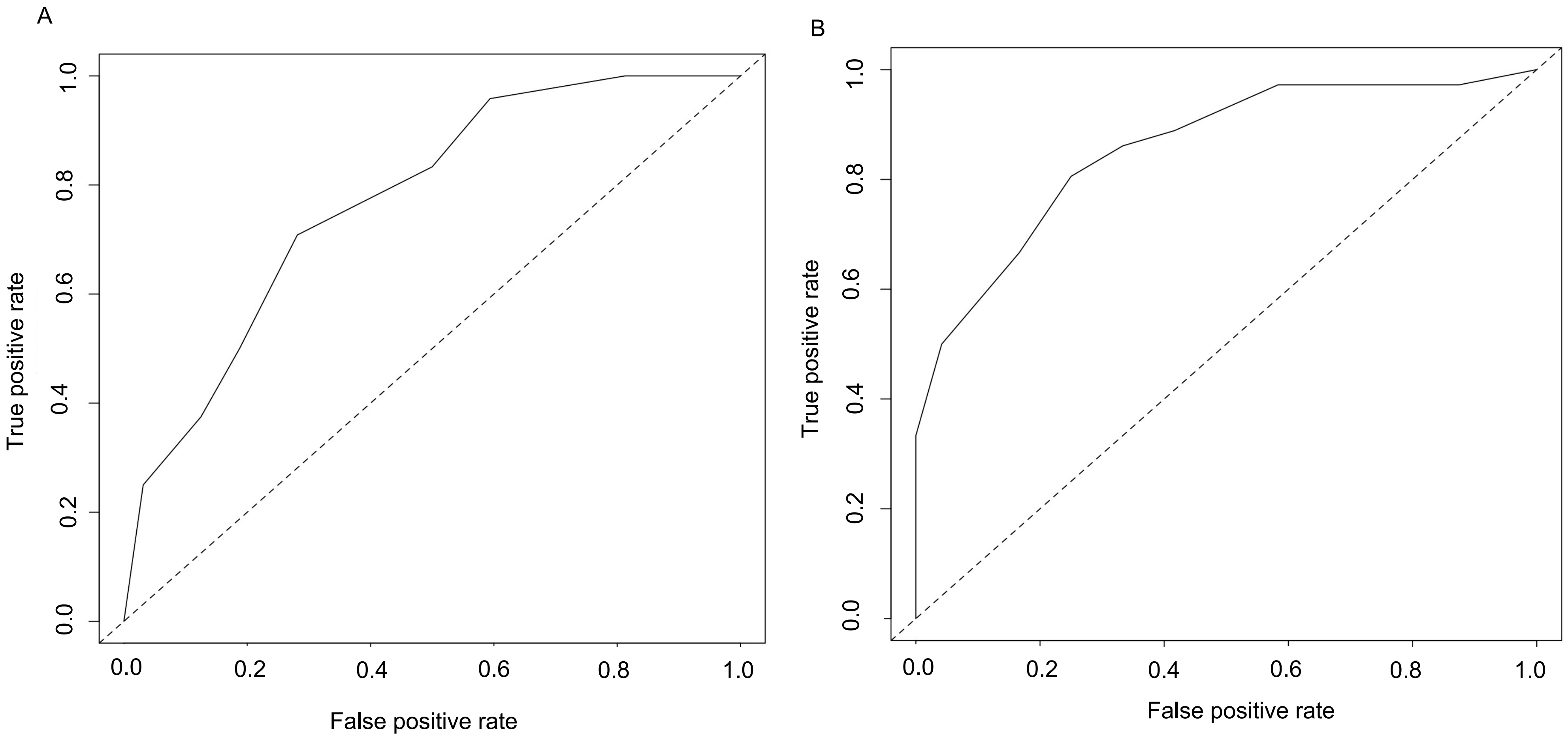

Figure S1: Receiver operating characteristic curve (ROC) indicating false and true positive rates of decision tree (DT) models. A) ROC curve of DT1, B) ROC curve for DT2.
