# Supplementary material for: Data science approaches provide a roadmap to understanding the role of abscisic acid in defence
Source: Quant Plant Biol. 2023 Feb 8;4:e2. doi: 10.1017/qpb.2023.1 (PMC10095806; doi:10.1017/qpb.2023.1)
Supplement: Supplementary file 1 [file S2632882823000012sup001.zip › S2632882823000012sup002.pdf]

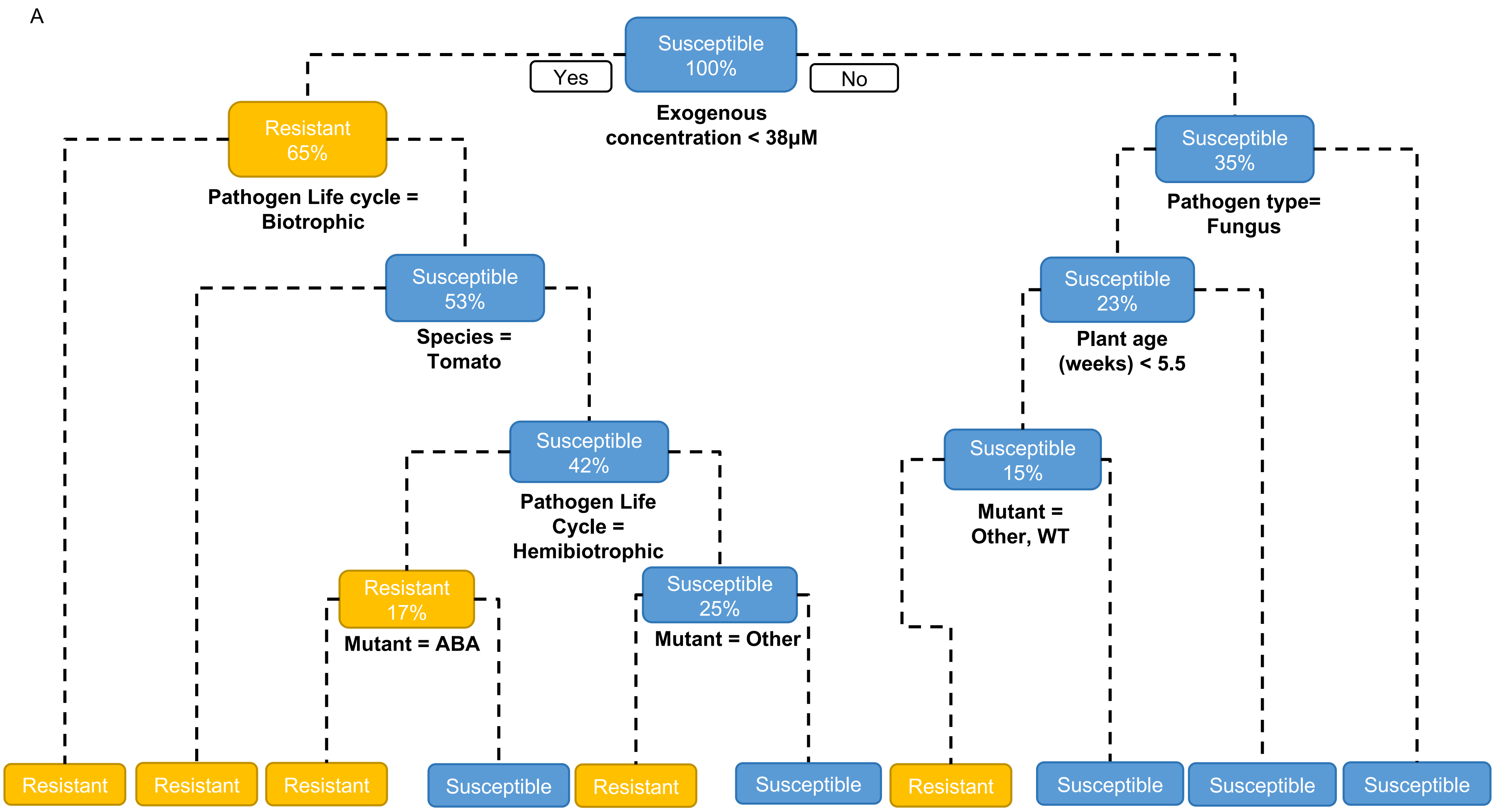

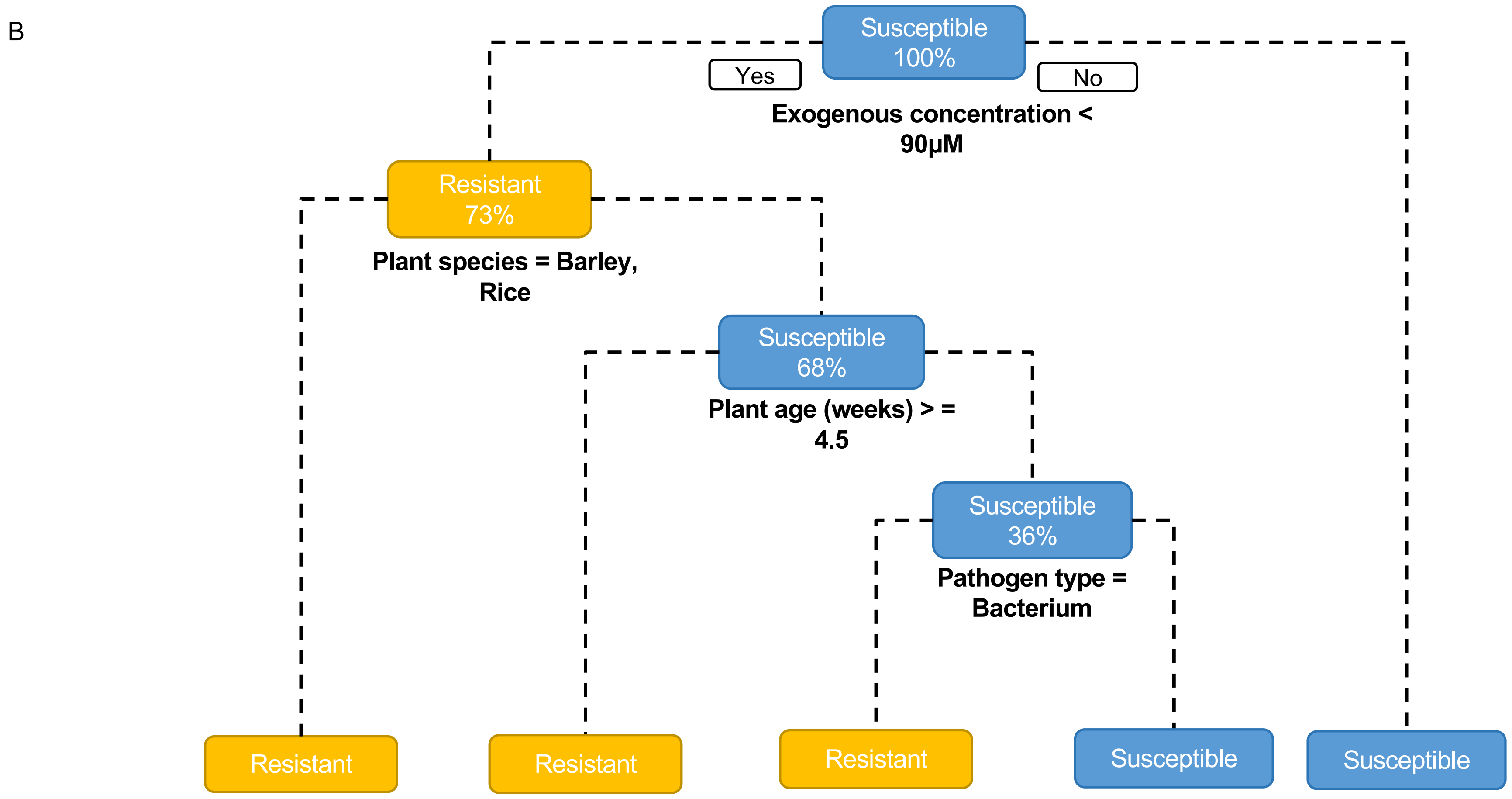

Figure S2:

Decision trees with minimised datasets predicting a binary resistance response of 'Susceptible' or 'Resistant'. (A) DText with extreme concentrations of exogenous ABA removed from the dataset, (B) DTage with plant ages over 6 weeks removed from the dataset . Yellow boxes indicate resistance and blue boxes indicate susceptibility. Percentage number in box represents proportion of dataset remaining following node split. DT is read from top root node to bottom leaf nodes, if the question asked at a node is 'yes', the left side is followed.
